# Primary hepatocytes as targets for Hepatitis C virus replication

**DOI:** 10.1111/j.1365-2893.2008.01051.x

**Published:** 2008-12

**Authors:** M J Farquhar, J A McKeating

**Affiliations:** Hepatitis C Research Group, Division of Immunity and Infection, University of BirminghamUK

**Keywords:** hepatitis C, hepatocyte, internalization, lipoproteins, receptors

## Abstract

Much of our current understanding of hepatits C virus (HCV) replication has hailed from the use of a small number of cloned viral genomes and transformed hepatoma cell lines. Recent evidence suggests that lipoproteins play a key role in the HCV life cycle and virus particles derived from the sera of infected patients exist in association with host lipoproteins. This report will review the literature on HCV replication in primary hepatocytes and transformed cell lines, focusing largely on host factors defining particle entry.

## Introduction

Hepatitis C virus (HCV) is a positive stranded RNA virus classified in its own genus, Hepacivirus, within the Flaviviridae family. Approximately, 170 million individuals are infected with HCV worldwide. The acute phase of infection is often subclinical with 70% of infected individuals developing a chronic infection leading to progressive liver pathology. The only established treatment is interferon-α, in combination with ribavirin, which is only partially effective, hence there is an urgent need for the development of more effective therapies. A full understanding of the pathogenesis of HCV requires the availability of tissue culture models that sustain viral replication and produce infectious particles. We will review the literature utilizing primary hepatocytes to study HCV infection and highlight commonalities and differences with the increasing literature using transformed hepatoma cell lines. This review focuses largely on viral entry and is restricted in scope to recent developments.

Much of our current understanding of HCV replication has been derived from the use of genomic replicons, which do not allow viral entry or assembly to be studied (reviewed in [1]). Dissection of HCV entry has been made possible using HCV pseudoparticles (HCVpp) [2,3]. The recent discovery that a genotype 2a strain of virus, derived from a *J*apanese patient with a rare case of fulminant hepatitis and named JFH-1, is able to replicate and release infectious particles in cell culture (termed HCVcc) has allowed studies on the complete viral life cycle [4–6]. Importantly, HCVcc is infectious in chimpanzees and virus recovered from infected serum remains infectious for hepatoma cells [7]. HCV infection is limited to humans and chimpanzees, imposing ethical, economic and technical limitations for *in vivo* experimentation. Although transplantation of immunodeficient mice with human hepatocytes generates mice with chimeric human livers which support HBV and HCV replication, allowing limited *in vivo* infection studies [8,9], the majority of experiments to date have involved the infection of cultured liver cells.

Techniques to study HCV entry have demonstrated the involvement of at least three host cell molecules, the tetraspanin CD81 [10,11], scavenger receptor BI (SR-BI) [12–14] and the tight junction protein family members Claudin-1, 6 and 9 (CLDN1, CLDN6 and CLDN9) [15–18]. Other molecules implicated in HCV entry are the low-density lipoprotein receptor (LDLr), Lipoprotein lipase [19], heparin sulphate and the mannose binding lectins L-SIGN and DC-SIGN (reviewed in [20]).

### HCV and lipoproteins

There is increasing evidence that lipids and lipid receptors are important in HCV infection. HCV isolated from patient serum (HCVser) is associated with lipoproteins and entry into hepatocytes has been suggested to involve lipid receptors. The majority of infectious viruses in the peripheral blood circulate in association with apolipoprotein B (ApoB) and apolipoprotein E (ApoE) [21,22]. The buoyant density of HCVser is heterogenous and particles have been isolated over a range of densities from 1.03 to 1.25 g/mL with the peak infectivity, as determined from animal challenge studies, in the lower density fraction(s). Extracellular HCVcc particles have also been reported to have a heterogeneous range of buoyant densities, with the lower density forms representing very low-density lipoprotein (VLDL)-associated particles [7,23,24]. Interestingly, HCVcc recovered from infected animals displayed a lower buoyant density and subsequent propagation of the virus in hepatoma cell lines resulted in a transition to higher buoyant density [4,7]. Moreover, the specific infectivity of HCVcc recovered from infected animals was higher than the input inocula, suggesting that virus association with lipoproteins increases or preserves the infectivity of low-density fractions.

Low-density lipoviral particles (LVP) containing core and viral RNA have been reported to exist in association with immunoglobulins and host triglyceride rich lipoproteins [25]. More recently, the HCV envelope glycoproteins and apolipoproteins ApoB, ApoE, ApoCII and ApoCIII have been identified at the surface of LVPs [24,26]. Analysis of the lipid composition of lipoproteins and of purified LVPs suggests that LVPs are not simply aggregates of lipoproteins and viral particles. Moreover electron microscopic investigation of LVPs in plasma fractions corresponding to low-density lipoproteins (LDLs) show large spherical structures of 100 nm diameter, whilst LDLs are more homogenous and of 25 nm in diameter [25]. Andre *et al.* proposed that LVPs assemble in the endoplasmic reticulum of hepatocytes, as opposed to associating with lipoproteins in the circulation. The ability of anti-ApoB antibodies to precipitate 50% of HCV RNA containing particles from infected liver support this hypothesis [27].

Recent reports have highlighted a critical role of lipoprotein assembly and secretion in the HCV life cycle, where treatment of hepatoma cells with a microsomal triglyceride transfer protein (MTP) inhibitor or siRNA silencing of ApoB/E expression reduced the levels of both VLDL and HCV in the extracellular media, suggesting that viral secretion is dependent on VLDL assembly and/or release [23,28,29]. Furthermore, HCVcc has been reported to replicate in cytoplasmic membrane vesicles enriched with ApoB, ApoE and MTP, proteins known to be required for the assembly of VLDL [29].

### HCV infection of hepatocytes

Human hepatocytes are thought to be the primary target cell supporting HCV replication *in vivo*. HCVpp entry into both hepatocytes and Huh-7 hepatoma cells is pH-dependent [3,30]. Studies to elucidate the involvement of clathrin in HCVpp internalization have shown that Brefeldin A and chloroprimazine treatment(s) significantly reduce entry, suggesting that viral entry is pH- and clathrin-dependent in both cell types. However, establishing and maintaining cultures of primary human hepatocytes (PHH) that sustain HCVcc replication has proven difficult. Hepatocytes support only low levels of HCV replication, requiring PCR detection of negative strand viral RNA to verify active replication [5,31–34]. In contrast, immortalized human hepatocytes have been reported to support HCV replication to higher levels [35], however, this artificial system involves the cell immortalization by HCV core prior to their electroporation with full-length HCV RNA. Nevertheless, the authors report viral replication and the detection of viral encoded proteins, NS5A and E1, by immunogold labelling. Lazaro *et al.* reported the successful HCVser infection of human fetal hepatocytes (HFH), with the release of infectious virus in the culture media that was able to infect naïve target cells [36].

HCVser infection of PHH has provided important insights into how the virus may infect the liver [31,32], demonstrating a role for CD81 and LDLr in infection. As previously described for HCVcc infection of hepatoma cell lines [4,5], anti-CD81 and soluble CD81 (sCD81) are both capable of inhibiting HCVcc infection of PHH. In contrast, HCVser infection of PHH could not be inhibited by sCD81, although the virus remained sensitive to the neutralizing effects of anti-CD81 antibodies. The ability to block infection of both HCVcc and HCVser with anti-CD81 antibodies demonstrates an important role of CD81 during infection of PHH and hepatoma cell lines. Conversely, the inability of sCD81 to neutralize HCVser infection suggests difference(s) between particles isolated from patient serum and those generated in cell culture. The authors suggest that particle-associated lipoproteins may prevent E2 associating with CD81 and prior interaction(s) of HCVser with cell surface molecules prime CD81-dependent entry steps to occur. Several studies have reported that anti-CD81 antibodies neutralize infectivity after viral attachment to the target cell, consistent with CD81 acting as a co-factor or internalization receptor [37–39]. The inability of sCD81-sepharose to precipitate virus from infected plasma is consistent with this model [40]. Current data support a model where E1E2 epitopes on HCVser are masked by associated lipoproteins whilst these epitopes are exposed on HCVcc particles. It is likely that HCV binds the cell surface via glycosaminoglycans [41–44] or lipoprotein receptors (LDLr/SR-BI) which lead to conformational change(s) in the viral particle allowing the engagement of CD81 and CLDN1 co-receptors.

Several reports have suggested that HCVser can bind cells in an LDLr-dependent manner and that particle internalization correlates with LDLr activity at the cell surface [25,45]. Treatment of PHH with squalestatin and 25-hydroxycholesterol to increase or decrease LDLr expression, respectively, also implied an association between LDLr expression and HCVser infection [31]. More significantly, antibodies specific for LDLr inhibited HCVser infection of PHH, lending further support for a role of LDLr in HCVser infection. A recent study analysed the binding of low-density HCV fractions from a liver macerate to the HepG2 hepatoma cell line [42]. Culturing cells in lipoprotein deficient medium and insulin increased LDLr expression and virus association, leading the authors to conclude that LDLr is the major receptor defining low-density HCV binding to cells [42]. In contrast, LDLr is not required for HCVpp entry [3,12], however, pseudoparticles are generated in 293T embryonal kidney cells that do not produce VLDL and are therefore unlikely to associate with lipoproteins and to mimic native HCV.

Conversely, Maillard *et al.* reported that Chinese hamster ovary cells engineered to over-express human scavenger receptor BI (SR-BI) bind HCVser via the ApoB moiety of VLDL [46]. SR-BI binds high density lipoprotein (HDL), although it also recognises LDL, VLDL and oxLDL. Antibodies specific for SR-BI have been reported to inhibit HCVcc infection [13,39,47,48]. Exogenous HDL promotes HCVcc and HCVpp infection via SR-BI [39,49–52] but has minimal effect(s) on HCVser infection of PHH [31]. Discord between HCVser and HCVcc interaction with SR-BI and LDLr may simply reflect their differential association with lipoproteins. However, some reports with HCVser measure virus binding rather than productive replication, making definitive interpretations of the *in vivo* relevance difficult. In summary, these data suggest that HCVser infection of hepatocytes is dependent on lipoprotein receptors, lending further support to the model that HCVser particles may differ significantly from HCVcc in their association with lipoproteins.

Sustained transmission of infectious HCV in HFH may offer the closest model to viral replication in the liver reported to date [36]. Lazaro *et al.* demonstrated HCV replication by quantifying viral genomes and by the detection of viral encoded structural and non-structural proteins. Whether transfected with HCV RNA or infected with HCVser, HFH exhibit distinct fluctuations in the amount(s) of virus released, a trend that has also been observed in infected chimpanzees and in HCVcc infected Huh-7.5 cells [4,7,36]. Infected HFHs demonstrate punctuate cytoplasmic NS3 staining in distinct areas or foci of infection, in agreement with recent reports of HCVcc infected Huh-7.5 cells [53]. After 5 days, HFHs release virus particles of heterogenous density with a major peak at 1.12 g/mL, however, by 40 days post-infection, the distribution was more homogenous and the peak of HCV RNA associated with a density of 1.17–1.18 g/mL, comparable to serum-derived and HCVcc particles. Electron microscopic examination of the extracellular media identified virus like particles of 50–90 nm in diameter that stained for E2 glycoproteins. This is the first report of hepatocytes supporting virus replication where viral proteins can be visualized and the cells exhibit some cytotoxicity following prolonged infection.

### Receptor expression

The receptors that have been implicated in HCV infection are not exclusively expressed within the liver. CD81 and CLDN1 are expressed in many tissues [54] although high levels of CLDN1 are found within the liver [55]; SR-BI is present in macrophages, steroidogenic and liver tissue [56]. Expression of the recently described receptor molecules CLDN6 and CLDN9 is also not restricted to the liver [16,18]. Hepatocytes in tissue and *ex vivo* express CD81, CLDN1 and SR-BI, consistent with their ability to support HCV infection [57]. The lack of hepatocyte restricted expression of the three receptor molecules suggests that their organization in hepatocytes may be important for viral receptor activity or that additional receptor molecules await identification. Our recent studies using FRET methodologies to detect CD81-CLDN1 association demonstrated protein complexes at the plasma membrane surface of both hepatocytes and Huh-7.5 hepatoma cells [58]. Receptor complexes were detected independent of virus infection and the presence of CD81/CLDN1 co-receptor complexes does not indicate viral permissivity [58].

### Polarization

The discovery by Evans *et al.* that the tight junction protein CLDN1 is a co-receptor for HCV highlighted the importance of studying cellular polarity in HCV entry [15]. Tight Junctions (TJs) are essential for establishing cell polarity and form a barrier to regulate paracellular transport of solutes across an epithelium. Hepatocytes are highly polarized, with their plasma membranes being separated by tight junctions into apical (canalicular) and basolateral (sinusoidal) domains [59,60]. In culture, hepatocytes rapidly lose polarity due to loss of tight junctional integrity, many methods are currently under investigation to promote and maintain hepatic polarity *in vitro* (reviewed in [60,61]). Several studies have been carried out to find a way to keep the hepatocytes isolated from normal liver in a well-differentiated and polarized state. For example, hepatocyte culture on and in various matrices can help to preserve their polarized functional features [62]. Polarization *in vitro* is slow and variable and as hepatocytes do not divide and differentiate their ability to establish polarity is compromised. However, the requirement of polarized cells and functional tight junctions for HCV entry awaits confirmation.

Mee et al. demonstrated that the epithelial cell line Caco-2, which express CD81, CLDN1 and SR-BI, supported HCV replication [63]. The viral receptors localized at the lateral cell–cell junctions and disruption of TJs enhanced HCV infection, suggesting that TJs may hinder viral access to receptors expressed on the lateral and basolateral domains. In Huh-7.5 hepatoma cells CD81 and CLDN1 co-localized predominantly at intercellular junctions, however, these cells were unable to form a barrier to solutes and disruption of TJs had no effect on HCV infection or TJ protein localization, suggesting minimal polarization [58,60,63]. This is in contrast to a recent report by Yang and colleagues [17], demonstrating that Huh-7 cells had a low level trans-epithelial resistance, consistent with polarization. These differences may simply reflect heterogeneity in Huh-7 clones between different laboratories. Current data suggests that functional TJs are not required for viral receptor activity and polarization of the cell reduces CD81-CLDN1 association and receptor activity (Mee & Harris, unpublished data). *in vivo* HCV may target hepatocytes where cell–cell contacts are damaged by inflammatory mediators or physical injury, allowing more efficient viral entry. This hypothesis is supported by the high levels of viral replication observed following liver transplantation, which may partly be a consequence of liver damage during surgery [64]. It is important to acknowledge that cultured PHH and Huh-7 cells exhibit negligible markers of polarization [60], however, the majority of hepatocytes *in vivo* will be polarized.

In polarized hepatocytes, the lipoprotein receptor SR-BI functions as an endocytic receptor mediating the uptake of lipoprotein cholesterol and secretion into bile [65]. Studies using the WIF-B cell model for polarized hepatocytes demonstrate SR-BI at the basolateral surface, ideally located for binding of lipoproteins and virus from the sinusoidal blood. Once the receptor is loaded with cholesterol, it transcytoses to the bile canaliculi at the apical surface of the polarized cell [66].

Earlier this year, we reported the co-localization of CD81 and CLDN1 at both the apical-canalicular and basolateral-sinusoidal hepatocyte surface(s) [57], whilst SR-BI co-localized with CLDN1 at the basolateral surface. FRET analysis using receptor specific antibodies supported an association between CD81 and CLDN1 in hepatocytes within human liver tissue. These studies suggest that CLDN1 exists in both tight junctional and non-junctional forms and, given the highly permissive nature of non-polarized Huh-7 cells, we suggest that HCV may preferentially utilise the non-junctional forms of CLDN1 to enter cells.

## Conclusions

The advent of HCVcc has enabled studies on the complete viral life cycle and the next major challenge is to unify observations made in different experimental systems with the goal to define mechanisms of HCV tropism and replication in the infected host. It will be important to extend current studies to include additional virus strains and hepatic cells to ensure the validity of our observations. In the absence of an immune competent small animal model that allows for the application of reverse genetics, serum-derived HCVcc recovered from infected animals will provide an interesting source of virus that may mimic the virus-lipoprotein complexes circulating in infected patients and will replicate efficiently *in vitro*.

## Abbreviations

ApoB: apolipoprotein B
HCV: Hepatitis C virus
HCVpp: HCV pseudoparticles
HDL: high density lipoprotein
HFH: human fetal hepatocytes
LDLp: low-density lipoviral particles
LDLr: low-density lipoprotein receptor
MTP: microsomal triglyceride transfer protein
PHH: primary human hepatocytes
TJs: tight junctions
VLDL: very low-density lipoprotein
